# Aggregation of germlings is a major contributing factor towards mycelial heterogeneity of *Streptomyces*

**DOI:** 10.1038/srep27045

**Published:** 2016-05-31

**Authors:** Boris Zacchetti, Joost Willemse, Brand Recter, Dino van Dissel, Gilles P. van Wezel, H. A. B. Wösten, Dennis Claessen

**Affiliations:** 1 Microbial Biotechnology, Institute of Biology, Leiden University, Sylviusweg 72, 2333 BE Leiden, The Netherlands; 2 Microbiology, Department of Biology, Utrecht University, Padualaan 8, 3584 CH Utrecht, The Netherlands

## Abstract

Streptomycetes are filamentous bacteria that produce numerous valuable compounds, including the majority of clinically used antibiotics. At an industrial scale, most of these compounds are produced in bioreactors. Growth of streptomycetes under these conditions is characterized by the formation of complex mycelial particles, whose sizes follow a bimodal distribution. Given the correlation between specific productivity and morphology, this size heterogeneity poses a potential drawback in industry. Recent work indicates that mycelial morphology is controlled by a number of genes that encode proteins required for the synthesis of cell surface-associated glycans. Using a quantifiable system based on fluorescent markers, we here show that these glycans mediate aggregation between germlings and young mycelia, yielding mycelial particles that originate from many different individuals. We also demonstrate that at later time points aggregation between distinct particles is no longer detectable. Notably, the absence of the corresponding glycan synthases yields mycelia that are homogeneous in size, identifying mycelial aggregation as a driving factor towards size heterogeneity. Given that aggregation is widespread within streptomycetes and can also occur between different *Streptomyces* strains, our work paves the way to improve *Streptomyces* as a cell factory for the production of known metabolites, but possibly also to discover new ones.

Streptomycetes are filamentous bacteria that are renowned for their ability to produce several types of bioactive compounds. Standing out among these are a plethora of antibiotics, but also numerous antiviral compounds, anticancer agents and molecules that suppress the immune system^1^,^2^. Due to their saprophytic lifestyle, streptomycetes also show a remarkable capacity to produce and efficiently secrete various hydrolytic enzymes that allow them to degrade almost any naturally occurring polymer^3^. The extensive industrial use of streptomycetes does not come as a surprise when these factors are taken into account.

In contrast to most other bacteria, streptomycetes grow by forming thread-like cells called hyphae^4^. These hyphae extend at their tip, while new hyphae emerge via subapical branching of pre-existing ones. This mode-of-growth leads to the construction of complex networks of interconnected hyphae called mycelia. When grown in liquid, these mycelia display markedly different morphologies^5^. Some strains, including the industrial workhorse *Streptomyces lividans*, predominantly form dense mycelial particles called pellets. In contrast, other strains form so-called mats, which are loosely entangled and scarcely dense mycelial networks^5^. Notably, a strong correlation exists between morphology and productivity in streptomycetes^5^,^6^,^7^. While growth as pellets is preferred for the production of antibiotics, it is suboptimal for the production of enzymes. For efficient enzyme production mycelial mats are more suitable, which relates to the fact that nutrients are more easily accessible to all hyphae. In fact, hyphae in the central part of pellets often suffer from nutrient stress leading to a phase of controlled cell death^8^,^9^. To further complicate matters, recent work from our lab demonstrated that liquid-grown *Streptomyces* cultures are heterogeneous and contain at least two populations of mycelial particles that differ in size^10^. This size heterogeneity was observed across a range of different streptomycetes and growth media, suggesting that it has a genetic basis.

In the past few years, several genetic determinants involved in mycelial architecture have been identified in *S. lividans*. These include among others the recently discovered *mat* genes^11^, as well as *cslA, glxA* and *dtpA*^12^,^13^,^14^. The common feature of these genes is that they encode proteins that are involved in the synthesis of extracellular glycans. Failure to synthesize these glycans abolishes pellet formation and leads to mycelia with an open, mat-like morphology^5^. This indicates that extracellular glycans play a pivotal role in shaping *Streptomyces* pellets, although the underlying mechanism is not known yet. One of the outstanding questions is whether these glycans solely mediate hypha-hypha interactions *within* individual particles or whether they also mediate aggregation of hyphae *between* distinct mycelia. The latter process, hereinafter referred to as aggregation, generates larger mycelial structures composed of different individuals. Aggregation has been well characterized in filamentous fungi, such as the ascomycete *Aspergillus niger*^15^. Here, aggregation was shown to be a two-step process that starts immediately after inoculation with the clustering of conidia. A second aggregation step coincides with the onset of germination, during which groups of germinated conidia aggregate to give larger particles. In streptomycetes, the clumping of mycelial particles has been noticed in *Streptomyces aureofaciens*^16^, although the extent of aggregation and factors involved in this process have remained obscure.

In this study we show that aggregation is restricted to the very early stages of growth, leading to the formation of mycelial networks that originate from different individuals. A qualitative and quantitative image analysis approach using fluorescent reporter strains demonstrated that aggregation depends on *cslA*, *glxA* and *matAB*. Excitingly, the absence of these genes yields mycelial particles that originate from individual spores, and which, as a consequence, leads to a homogeneously distributed population. We also show that aggregation in streptomycetes can occur between different wild-type species. Taken together, our data provides the basis to enhance the industrial exploitation of these prolific bacteria.

## Results

### A quantifiable system to analyse particle aggregation in *Streptomyces lividans*

**Figure 1:**
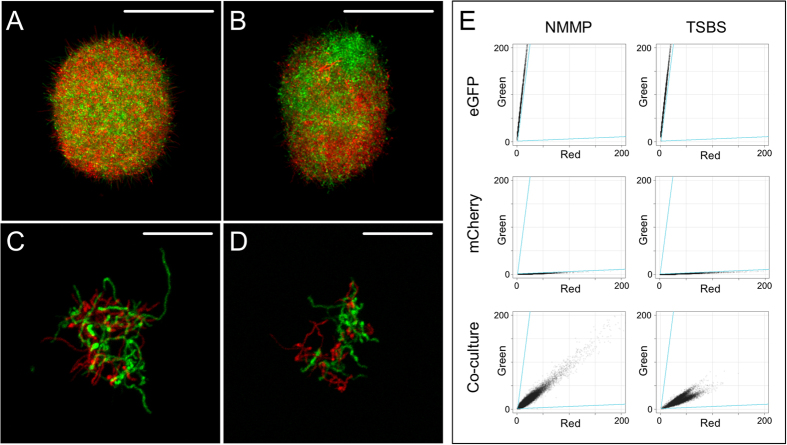
Analysis of aggregation of fluorescent *S. lividans* strains. Spores of strains expressing eGFP (*S. lividans* pGreen) or mCherry (*S. lividans* pRed) were mixed at the onset of growth in NMMP (**A**,**C**) or TSBS (**B**,**D**). After 24 h of growth, pellets are composed of both types of fluorescent hyphae (**A**,**B**). Aggregates of fluorescent germlings are already detected 6 hours after inoculation (**C**,**D**). (**E**) Quantitative analysis of aggregation using particle sorting. Each plot represents the fluorescence intensities of pellets in the red (X-axis) and green (Y-axis) channel. The top two panels show the green:red ratios of pellets from the eGFP-expressing strain in NMMP (left) and TSBS (right) medium, while the middle two panels reveal the green:red ratios of pellets from the strain expressing only mCherry. The bottom panels represent the fluorescence intensities of pellets from the co-cultivations of both fluorescent strains, which indicate that almost all pellets express both reporters. Scale bars represent 100 μm (**A**,**B**) or 20 μm (**C**,**D**).

To study aggregation in liquid-grown cultures, fluorescent strains of *S. lividans* 1326 were created that constitutively express eGFP or mCherry by placing these genes under control of the *gap1* promoter of *S. coelicolor*. The reporter strains were co-cultivated in TSBS or NMMP medium, resulting in the formation of pellets^10^. When spores of both fluorescent strains were mixed, pellets obtained after 24 h of growth were invariably composed of green and red fluorescent hyphae (Fig. 1A,B). Aggregates of fluorescent germlings were already visible 6 h after inoculation (Fig. 1C,D), indicating that aggregation is an early event (see below). Quantification of aggregation using the COPAS, a flow cytometer for large particles^17^, revealed that >99.8% of pellets contained both red and green fluorescent hyphae, irrespective of the used medium (Fig. 1E, Table 1).

### Aggregation is mediated via germlings

**Figure 2:**
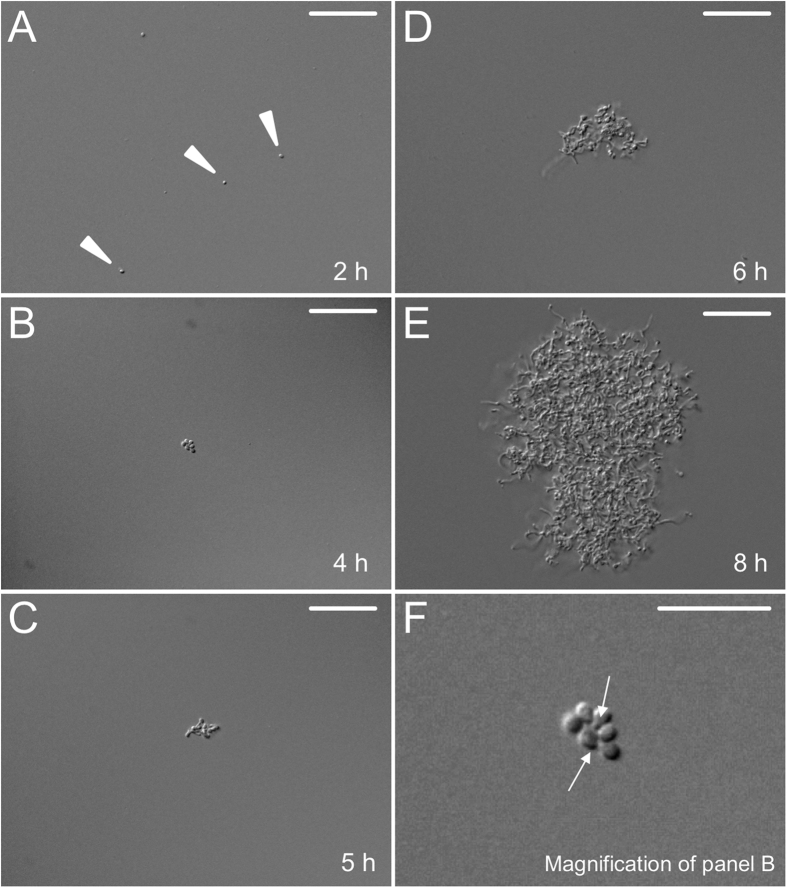
Microscopy analysis of early stages of growth and aggregation. Wild-type spores of *S. lividans* were inoculated in TSBS medium. Only individual spores (arrowheads) are observed 2 h after inoculation (**A**). Aggregation becomes visible after 4 h of growth (**B**) coinciding with the appearance of germ tubes (see also panel **F**). Further aggregation and growth leads to the formation of particles that increase in size over time (**C**–**E**). Panel (**F**) shows a magnification of the aggregate visible in panel (**B**). Arrows indicate germ tubes. Scale bars represents 20 μm (**A**–**E**) or 5 μm (**F**).

The early stages of aggregation of *S. lividans* were monitored using light microscopy (Fig. 2). In the first 2 h after inoculation, only individual spores were detected in the medium (Supplementary Fig. S1). Germination of spores after 4 h coincided with small aggregates that increased in size in time. Germ tubes were consistently visible in these aggregates (see magnification Fig. 2 and Supplementary Fig. S2), implying that they are required for aggregation. In agreement, individual spores that had not initiated germination were not part of aggregates (Supplementary Fig. S3).

**Figure 3:**
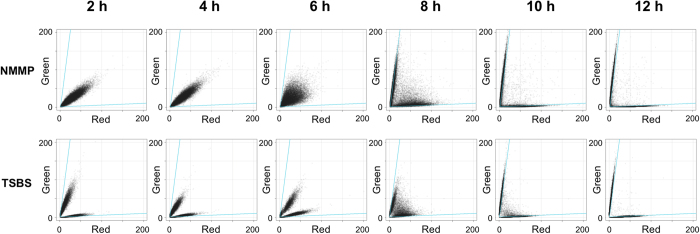
Quantitative analysis of time-dependent aggregation. Fluorescence profiles of 24-hour-old pellets obtained from co-cultures of *S. lividans* pRed and *S. lividans* pGreen that had initially been grown separately for 2, 4, 6, 8, 10, or 12 h. Each plot represents the fluorescence intensities of pellets in the red (X-axis) and green (Y-axis) channel determined by particle analysis. The growth media were NMMP (top) and TSBS (bottom). Note that aggregation is strongly reduced when the fluorescent strains have initially been grown separately for more than 8 h.

To more closely study whether aggregation specifically involves germlings or can also occur between mature mycelia, co-cultivations were performed of the red and green fluorescent strains that had been grown separately for 2, 4, 6, 8, 10 or 12 h. Analysis of the mixed cultures 24 h after the inoculation of spores indicated that virtually all pellets were composed of red and green fluorescent hyphae if the strains were mixed after 2, 4, 6 and 8 hours of growth (Fig. 3, Table 1). Conversely, when the strains were mixed after 10 or 12 h, aggregation became increasingly rare, as most pellets were only red or green fluorescent (Table 1). Notably, while pellets contained intertwined red and green fluorescent hyphae when the cultures were mixed after 2 h of growth, a patched pattern of green and red mycelial parts was observed in pellets when the cultures were mixed after 8 h of growth (Supplementary Fig. S4). To exclude that compounds accumulating in the culture medium reduced aggregation at late time points, we mixed washed mycelia of the fluorescent strains in fresh medium after 12 hours of growth. After washing, 3.5 ± 0.6% of particles aggregated in NMMP medium, while in TSBS medium 16.1 ± 6.1% of particles contained both fluorescences (Supplementary Fig. S5). Given that these values are similar to those observed without washing, these data indicate that aggregation is not hampered by compounds accumulating in the culture broth.

### Genes involved in glycan synthesis are required for germling aggregation

**Figure 4:**
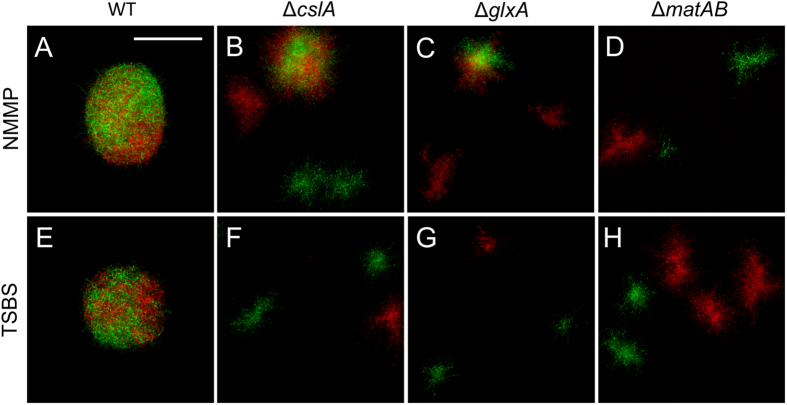
Aggregation depends on glycan synthases. Microscopy images of particles obtained 24 h after mixing spores of red- or green-fluorescent derivatives of the wild-type (WT), *cslA, glxA* or *matAB* mutant strains. Note that the majority of particles only have either green or red fluorescence in the absence of *cslA*, *glxA,* or *matAB* in both NMMP (top) and TSBS (bottom). Scale bar represents 100 μm (**A**,**E**) or 200 μm (**B**–**D**, **F**–**H**).

Given the crucial role of extracellular glycans in pellet morphology, we studied whether these polymers also mediate germling aggregation. We therefore created reporter strains of the *S. lividans cslA, glxA* and *matAB* mutants, all of which are affected in glycan biosynthesis^11^,^12^. Aggregation of the *cslA* and *glxA* mutants was strongly affected in both NMMP and TSBS, as indicated by the presence of particles that only showed green or red fluorescence (Fig. 4, Supplementary Figs S6–S9). Quantification revealed that 20.2 ± 3.6% and 3.5 ± 0.7% of the particles of the *cslA* mutant contained both red and green fluorescent hyphae in NMMP and TSBS medium, respectively. These values were comparable to those of the *glxA* mutant, where 23.0 ± 3.9% (TSBS) and 0.57 ± 0.2% (NMMP) of all particles were aggregated. Aggregation in the *matAB* mutant was also greatly reduced (Fig. 4), with only 4.8 ± 1.7% (NMMP) and 2.3 ± 0.2% (TSBS) of particles displaying both red and green fluorescent hyphae. These results indicate that proteins involved in glycan biosynthesis play a crucial role in the aggregation of germlings.

Considering that the majority of aggregation occurs within the first 12 h of growth (see above), we asked whether expression of the genes involved in glycan biosynthesis was changed after 12 h of growth. We therefore isolated RNA from mycelia grown in NMMP and TSBS medium after 12 and 24 h and performed RT-PCR using primers directed against *cslA, glxA, matA* and *matB*. Expression of these genes was detected in both media at both time points (Supplementary Fig. S10). This implies that the reduced aggregation after 12 hours is probably not caused by changes in the abundance of these glycan synthases.

### Germling aggregation leads to size heterogeneity in a medium-dependent manner

**Figure 5:**
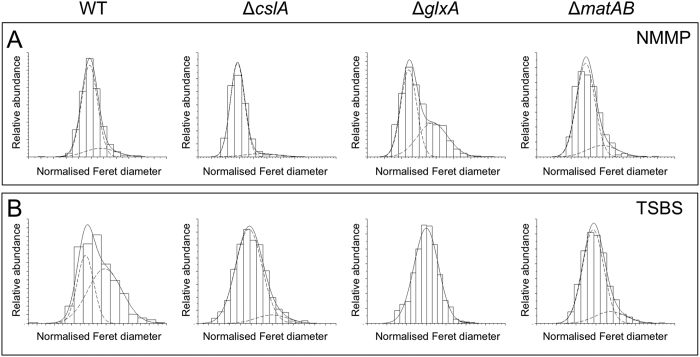
Quantitative analysis of particle size heterogeneity in the absence of *cslA, glxA* and *matAB*. Normalized particle size distributions of the *S. lividans* wild-type strain, the *cslA* deletion strain, the *glxA* deletion strain and the *matAB* deletion strain in NMMP (**A**) and TSBS (**B**) after 12 h of growth. The x-axis represents the normalized Feret diameter, while the y-axis represents the relative abundance. The two dashed lines in each plot represent the two normally distributed populations of particles fitted to the actual measured distribution (solid line). Size heterogeneity is strongly diminished in the absence of the *cslA*, *glxA* and *matAB* genes in TSBS medium.

Mycelia of the wild-type strain and its *cslA*, *glxA* and *matAB* mutant derivatives were analysed using automated image analysis to assess how aggregation affects the size distribution of mycelial particles. This approach enabled the analysis of particles that are too small for detection with the COPAS (see Methods). Mathematical analysis revealed that the size distributions of all strains grown for 12 h in NMMP was better explained by assuming the presence of at least two populations of particles that differ in size (Fig. 5A, Table 2). The size distribution of the wild-type strain was very similar to that of the *cslA* and *matAB* mutants, in which the number of small particles exceeded that of the bigger particles, as deduced from the skewed participation fractions (Table 2). In contrast, the *glxA* mutant in NMMP medium was more heterogeneous in size, with 60.7% of the particles being part of the population of small particles. Notably, the degree of heterogeneity in the size of wild-type pellets was considerably higher in TSBS medium compared to NMMP (Fig. 5B). This is concluded from the fact that in this case 38% and 62% of the particles belonged to the population of small and large particles, respectively (Table 2). In contrast, TSBS-grown cultures of the *cslA, glxA* and *matAB* deletion strains revealed a dramatic reduction in particle size heterogeneity. In all cases, the population of small particles accounted for the majority of mycelia in these cultures, with participation fractions of 93%, 99% and 84% for the *cslA, glxA* and *matAB* deletion strains, respectively (Table 2). Taken together, these results demonstrate that aggregation leads to heterogeneity in a medium-dependent manner.

### Mycelial aggregation of different streptomycetes yields multi-species pellets

**Figure 6:**
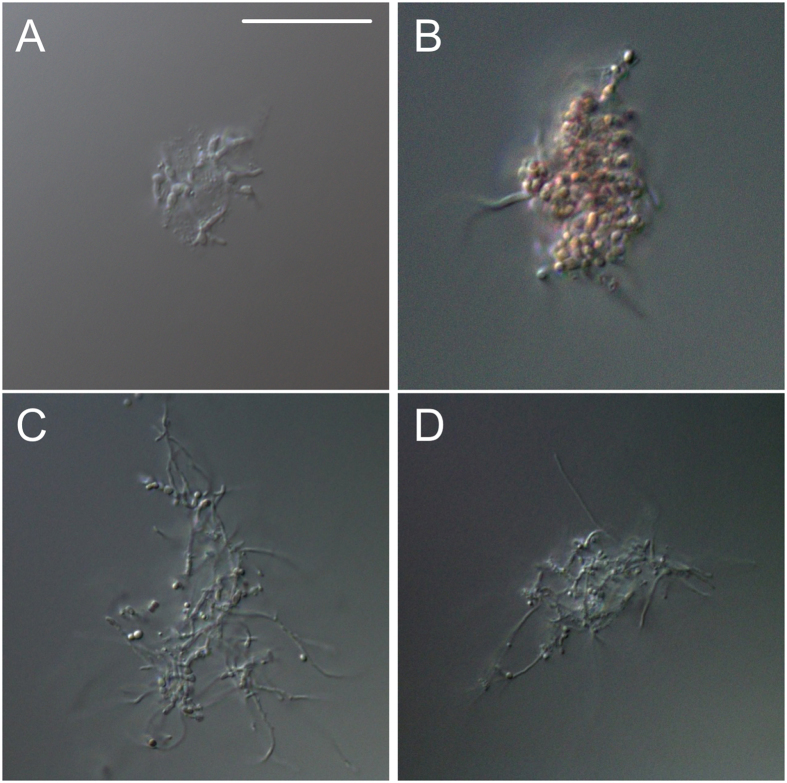
Germling aggregation is common in *Streptomyces.* Microscopy images of aggregates of *Streptomyces lividans* (**A**), *Streptomyces coelicolor* (**B**), *Streptomyces scabies* and *Streptomyces albus* (**D**) taken 6 h after inoculation in NMMP. Scale bar represents 10 μm.

**Figure 7:**
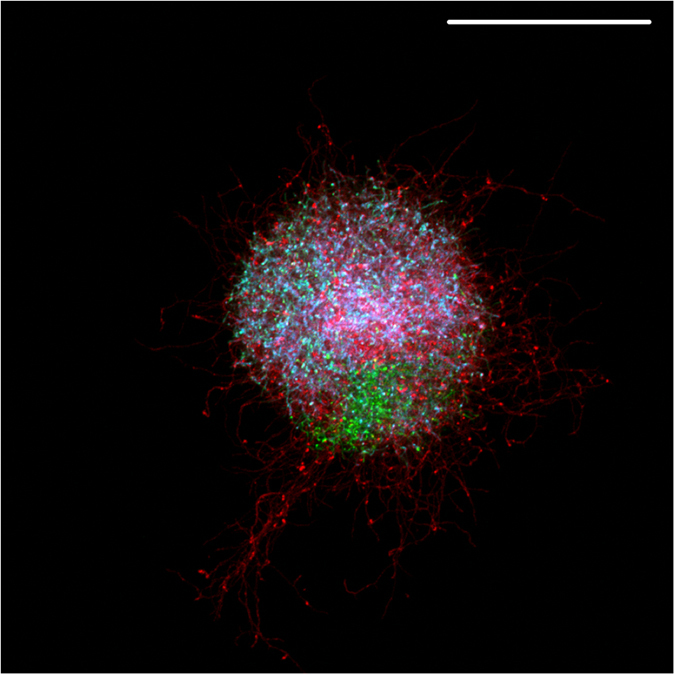
Formation of multi-species *Streptomyces* pellets. Germlings of *S. coelicolor* M512 (green), *S. scabies* (red) and *S. lividans* (false-colored in cyan) aggregate to form multi-species pellets. The image shows a representative pellet obtained after 24 h of growth of the three strain in NMMP medium. Scale bar represents 100 μm.

To determine whether aggregation is a common trait in streptomycetes, spores of different wild-type strains, i.e. *S. lividans, S. coelicolor, S. scabies* and *S. albus,* were inoculated in NMMP medium, after which the cultures were analysed via light microscopy. Aggregates of germinated spores were detected in all strains 6 h after inoculation (Fig. 6). In the case of *S. lividans* and *S. coelicolor* the aggregates appeared rather compact, unlike those of *S. scabies* and *S. albus* that had a more open morphology. To assess whether aggregation also occurs between different species, fluorescent wild-type strains of *S. lividans*, *S. coelicolor* and *S. scabies* were co-cultured in NMMP and in TSBS. The strains of *S. coelicolor* and *S. scabies* used in this experiment expressed eGFP and mCherry respectively, whereas *S. lividans* was engineered to produce both fluorescent proteins, and was false-colored cyan. Notably, all pellets after 24 h of growth were composed of the three different strains, irrespective of the medium (Fig. 7). These results not only indicate that germling aggregation is common in streptomycetes, but also shows that it occurs between different *Streptomyces* species.

## Discussion

Streptomycetes are among the biggest players in the fermentative production of antibiotics, hydrolytic enzymes and numerous other compounds with biological activity. The hallmark of the submerged growth of these microorganisms, the preferred mode of growth under industrial settings, is the formation of interconnected clumps of mycelium with varying sizes^10^,^18^,^19^,^20^. The constraints imposed by this heterogeneity are most evident when one takes account of the strong link between morphology and production, which is a well-established concept in the *Streptomyces* field^5^,^6^,^7^. We here show that the aggregation of germlings is an important factor leading to size heterogeneity. Our findings open new avenues to improve *Streptomyces* as a cell factory in the biotech industry.

### Aggregation in *Streptomyces*

In this work we present direct evidence for aggregation of independent particles in liquid-grown *Streptomyces* cultures. Whereas we mainly focus on the model strain *S. lividans* to provide insight into this phenomenon, we show that aggregation is common to a wide range of streptomycetes, and also occurs between the mycelia of different co-cultured *Streptomyces* species. About two-thirds of all *Streptomyces* genomes contain the *matAB* genes^11^, including all species used in this work, whereas the *cslA/glxA* operon is present in all streptomycetes^21^. The broad conservation of these genes across the streptomycetes could explain why aggregation of germlings is so common within this genus. Unlike in filamentous fungi such as *Aspergillus niger* and *Aspergillus fumigatus*^22^,^23^, in streptomycetes we did not find evidence for aggregation of ungerminated spores. Spores of streptomycetes are covered with amyloid fibrils that render the spore surface hydrophobic^24^,^25^. Apparently, this hydrophobicity is not a driving factor for aggregation, as aggregation was only detected after the visible emergence of germ tubes. The proteins that make up these amyloidal structures at the spore surface, called rodlins and chaplins, were previously shown to mediate attachment to abiotic surfaces^25^,^26^.

As aggregation of germlings proceeds, aggregates may also connect to one another leading to the formation of larger mycelial particles. Interestingly, the greater part of aggregation takes place between 4 and 10 h after the inoculation of the spores. These aggregation dynamics are in line with observations performed in a pioneering study on *S. aureofaciens,* which displayed aggregated filaments after approximately 8 h of growth^16^. Given that aggregation is not impeded by molecules accumulating in the culture medium and that the glycans involved in aggregation are constitutively produced, we assume that this time-restricted process is caused by the size of the interacting particles, which may become too large to remain connected due to increasing collision forces or shear stress imposed upon them.

As shown in this work, for aggregation to occur between different germlings, extracellular glycans associated with the cell surface are of crucial importance. Notably, aggregation in filamentous fungi is also mediated by glycans. In *A. fumigatus*, for instance, an α-(1–3) glucan becomes exposed upon swelling of spores and mediates conidial aggregation^22^. In *S. lividans*, the glycans produced by CslA and MatAB are key contributors mediating aggregation. The most dramatic phenotype in the absence of *cslA* and the *matAB* genes is observed in TSBS-grown cultures, where aggregation was largely abolished in both deletion mutants. Conversely, in NMMP-grown cultures aggregated particles were detected, although significantly fewer than for the parental strain. These results suggest that other adhesives may contribute to aggregation in NMMP-grown cultures. Differences in the composition of a so-called extracellular matrix under various conditions is in fact not uncommon, and has also been observed in other bacteria during the formation of biofilms^27^,^28^. A wide range of molecules normally composes the extracellular matrix of bacteria, which in addition to glycans may contain proteins, lipids and nucleic acids^4^,^29^,^30^,^31^. In this respect it is interesting to mention that pellet formation resembles the formation of a free-floating biofilm or floc^14^,^32^. In agreement with this, a previous report suggested that extracellular DNA is involved in maintenance of pellet morphology in *S. coelicolor*^33^. Besides providing the first direct indication of aggregation between distinct particles in streptomycetes, our work also demonstrates that this phenomenon can occur between different *Streptomyces* species, resulting in the formation of multi-species pellets. These “synthetic” communities may be useful for the discovery of novel metabolites. Several recent examples indicate that co-cultivations may lead to increased levels of known secondary metabolites, but also to the production of novel compounds^34^,^35^,^36^,^37^. Notably, physical adhesion was found to be essential to elicit the biosynthesis of interaction-specific compounds in co-cultures of *Aspergillus nidulans* and *Streptomyces hygroscopicus*^34^. We anticipate that the use of aggregation between germlings of different streptomycetes may represent a useful strategy to discover novel compounds. We are currently using this approach to study whether novel metabolites can be produced by these synthetic communities.

### Factors leading to size heterogeneity

Our results highlight the importance of extracellular glycans for the aggregation of independent particles, which ultimately provides the basis for mycelial size heterogeneity in *Streptomyces*. Germling aggregation leads to a range of particles that not only increase in size due to growth, but also due to the incorporation of germlings or small mycelia into pre-existing particles. In addition, larger-sized particles have an increased chance to capture new germlings by accidental collision, thereby further skewing the differences between small and large particles. In contrast, particles of the *cslA* and *matAB* mutant strains only increase in size due to growth, which may explain why they are more homogeneous in size. The fact that mycelia likely grow faster in rich medium can explain why pellets of the *S. lividans* wild-type strain are earlier heterogeneous in size in TSBS medium, compared to the minimal NMMP medium. However, we also cannot exclude that other surface-associated molecules that are differentially produced in both conditions may contribute to generating size heterogeneity.

One surprising outcome was the observation that the *glxA* mutant strain is heterogeneous in size in NMMP medium after 12 h of growth, while all other strains were homogenous. More specifically, the fraction of large particles in the *glxA* mutant was considerably larger compared to that of the *cslA* mutant (Fig. 5 and Supplementary Fig. S11). Notably, these larger particles were significantly denser than those being part of the population of small particles (Supplementary Fig. S11). One main difference between the *glxA* and *cslA* mutant is the fact that the *glxA* mutant is still capable of synthesizing the glycan produced by CslA, on which GlxA would normally act. Perhaps this residual glycan, although not processed by GlxA, is still capable of inducing some mycelial aggregation leading to the formation of denser particles that comprise the observed second distinct population.

Opposite to aggregation, another factor that directly influences size heterogeneity is fragmentation. By fragmentation we mean the detachment of viable mycelial parts from existing particles. This process could be particularly significant for strains such as the *cslA* and *matAB* mutants, which no longer form dense pellets, and as a consequence are more susceptible to shear stress. Such detached fragments directly lead to a change in the distribution of particles in the culture. By making use of the fluorescent strains described in this paper, we have gathered preliminary evidence for fragmentation (B. Zacchetti and D. Claessen, unpublished results). Whether this process already occurs within the 12-hour timeframe described here remains to be elucidated. We hypothesize that these combined factors provide the basis for the formation of two distinct populations of pellets. We are now building a mathematical model to gain more insight into how these complex processes lead to heterogeneity.

As mentioned previously, a strong link exists between mycelial morphology and production of secondary metabolites and enzymes in streptomycetes. The observation that the deletion of *cslA* and the *matAB* genes not only reduces the size of mycelial particles, but also their heterogeneity makes them interesting targets to improve enzyme production. In agreement with this, we recently showed that a *S. lividans* strain lacking the *matAB* genes produces more tyrosinase than the wild-type strain^11^. Given the propensity of many streptomycetes to aggregate and grow heterogeneously, we expect that targeting these processes may be valuable for production purposes in other *Streptomyces* species.

## Methods

### Strains and culture conditions

The *E. coli* and *Streptomyces* strains used in this study are listed in Supplementary Table S1. *E. coli* strains were grown at 37 °C in LB medium, supplemented with antibiotics if necessary. For growth of *E. coli* strains carrying a plasmid with an hygromycin resistance cassette, modified LB medium was used, which contains 1.25 gl^−1^ NaCl instead of 10 gl^−1^. *Streptomyces* strains were grown at 30 °C on solid MS agar plates. For growth in liquid, 250 ml flasks equipped with coils were used, each containing 100 ml TSBS medium (TSB + 10% sucrose) or NMMP medium with glucose as carbon source^38^. Cultures were inoculated with 10^6^ spores ml^−1^ (both when single or two strains were used) and were grown at 30 °C in an orbital shaker at 180 rpm.

### Constructs and transformation

Constructs and oligonucleotides used in this work are listed in Supplementary Table S2 and Supplementary Table S3, respectively. To create pGreen, the promoter region of *gap1* (SCO1947) of *S. coelicolor* A3(2) M145 was amplified via PCR as a BglII-NdeI 450 bp fragment using primers Gap1-FW and Gap1-RV. This promoter has a strong constitutive activity in *S. lividans* (Mangiameli and Vijgenboom, unpublished). The promoter fragment was ligated as a BglII-NdeI fragment into pIJ8630, containing the *eGFP* gene^39^. To create pRed, the integrative vector pMS82 was used, which carries an hygromycin resistance cassette^40^. The *gap1* promoter was amplified via PCR using primers Gap1-FW* and Gap1-RV, thereby introducing KpnI and NdeI sites. The *mCherry* gene was amplified as an NdeI-HindIII fragment using vector pRSET-B as a template [R. Tsien] and primers mCherry-FW and mCherry-RV. The two PCR products were ligated in vector pIJ2925^41^, after which the *gap1-mCherry* fragment was excised with KpnI and HindIII and blunted with T4 polymerase, according to the manufacturer’s protocol. The fragment was subsequently cloned into the EcoRV site of pMS82. Restriction analysis and sequencing confirmed the insertion and orientation of the fragment, with the *mCherry* coding sequence running in the opposite orientation relative to the hygromycin resistance gene. The reporter constructs were introduced in *Streptomyces* via conjugation as described^38^.

### RNA Isolation and RT-PCR

Total RNA was isolated in duplicate from mycelia grown for 12 or 24 h in TSBS or NMMP medium using the RNeasy Mini Kit in combination with the RNAprotect Bacteria Reagent (Qiagen), according to the instructions of the manufacturer. Following RNA extraction, 250 ng of RNA was used to generate cDNA followed by PCR amplification using the SuperScript III One-Step RT-PCR System (Invitrogen). The program for cDNA synthesis and amplification was as follows: cDNA synthesis (55 °C, 30 min); 30 cycles of 94 °C for 15 s (denaturation), 60 °C for 30 s (annealing), and 68 °C for 30 s (extension), followed by a final extension for 5 min at 68 °C. Specific primers for the RT-PCR are shown in Supplementary Table S3. As a control, primers directed against the 16S rRNA were used as described previously^42^.

### Flow cytometry

5 ml samples were harvested from liquid NMMP or TSBS cultures and fixed in 4% formaldehyde for 20 min on ice. Biomass was pelleted at 1500 rpm for 10 min, washed twice with phosphate-buffered saline (PBS), and stored at −20° until further use. Particles were analysed using a COPAS Plus profiler (Union Biometrica) equipped with a 1 mm nozzle as described^10^,^43^, excluding data with an extinction below 25, which correspond to hyphal fragments and debris. All experiments were performed in duplicate, and for each replicate at least 10,000 pellets were analysed per sample.

To calculate the number of pellets expressing both eGFP and mCherry, the green to red ratio was calculated of all pellets in the single cultivations of the fluorescent strains. The lowest value in the eGFP-expressing strain and the highest value in the mCherry-expressing strain were used as boundaries between which pellets were considered to express both reporter proteins. For fluorescence measurements, the photomultipliers were set at 800 and 900 Volts to detect the green and red signals, respectively. To compensate for the fluorescence bleed-through, the red signal was multiplied by a factor of 0.04 and subtracted from the green signal. The opposite was done for the red signal, with the green signal being multiplied by a factor of 0.05.

### Microscopy

Confocal microscopy was performed using a Zeiss Observer Microscope. Samples were excited with laser light at wavelengths of 488 and 543 nm to detect eGFP and mCherry, respectively. Fluorescence emissions were monitored in the region between 505–545 nm for eGFP, while a 560 nm longpass filter was used to detect mCherry. Capture settings were such that no bleed through could be detected. The pinhole size was adjusted to one airy unit (94 micron) for optimal contrast images which results in 4.5 μm thick coupes. All detailed fluorescence microscopy pictures (i.e. those in Figs 1, 4 and 7) represent Z-projections of stacks with a thickness of 2.2 μm (optimized for optical section thickness).

To quantify aggregation in mutants of *S. lividans*, whole microscopy wells (1 μ-Slide 2 × 9 well, Ibidi GmbH) containing samples of co-cultivations were imaged as a mosaic of 5 × 5 tiles. The eGFP and mCherry fluorescence emissions were merged to give 8-bit greyscale pictures that were used to detect regions containing fluorescent particles. The mean green and red fluorescence component were subsequently measured in the corresponding regions in RGB pictures. Data were used to quantify the number of particles showing green and/or red fluorescence. Particles with a green to red emission ratio between 1.2 and 0.15 were counted as aggregated, while those with a ratio above 1.2 or below 0.15 were counted as green or red, respectively. For each sample, at least 500 particles were analysed in duplicate. Examples of the images used for quantification are presented as Supplementary Figs S6–S9.

Light microscopy was performed using a Zeiss Axioscope A1 upright microscope with an Axiocam MRc5 camera at a resolution of 37.5 nm/pixel. Automated acquisition of pictures for size analysis of streptomycetes was performed using a brightfield Zeiss Observer microscope.

### Size distribution analysis

12 hours after the inoculation of spores, 10 ml samples were harvested from the culture flasks. The biomass was concentrated via centrifugation at 1000 rpm for 30 mins at 4 °C and resuspended in Phosphate Buffer Saline (PBS). Micrographs of samples were obtained via automated image acquisition and analysed using ImageJ (Joost Willemse, Ferhat Büke, manuscript in preparation). For size distribution analysis, the maximum Feret diameter was taken as the value representing the size of particles. For each measurement, 1000 particles were analysed to provide a sufficiently large statistical pool for further analysis. Incorrectly analysed particles (e.g. out-of-focus mycelia) were manually removed from the datasets. The size distribution analysis was performed as described^44^. Briefly, the datasets representing the normalized maximum Feret diameter of particles were fit by a probability distribution assuming two normal distributions. This model determines five parameters: the participation fraction (p), two means (μ1; μ2), and two standard deviations (σ1; σ2). For each parameter, the 95% confidence interval (CI) estimate was obtained by refitting with the model after bootstrapping (1000 replicates) using the open source Scilab language. Datasets with non-overlapping CIs of the mean and 0.025 < p < 0.975 were considered to be derived from a culture with two populations of pellets.

## Additional Information

**How to cite this article**: Zacchetti, B. *et al.* Aggregation of germlings is a major contributing factor towards mycelial heterogeneity of *Streptomyces*. *Sci. Rep.* **6**, 27045; doi: 10.1038/srep27045 (2016).

## Supplementary Material

Supplementary Information

## Figures and Tables

**Table 1:** Time-dependent aggregation of fluorescent strains in TSBS and NMMP medium.

Time (hours)	TSBS	NMMP
0	>99%	>99%
2	>99%	>99%
4	>99%	>99%
6	>99%	>99%
8	98.3 ± 0.89	93.0 ± 0.33
10	31.2 ± 1.3	29.1 ± 2.2
12	22.1 ± 7.8	10.6 ± 2.4

The values represent the percentage of particles expressing both fluorescences when mixed after 0–12 hours of growth.

**Table 2:** Participation fractions of the two populations of particles in liquid-grown cultures of *Streptomyces lividans* strains.

	NMMP		TSBS	
	Small particles	Large particles	Small particles	Large particles
WT	89.5 ± 7.1	10.5 ± 7.1	34.7 ± 4.2	65.3 ± 4.2
Δ*cslA*	94.9 ± 3.6	5.1 ± 3.6	93.2 ± 2.4	6.8 ± 2.4
Δ*glxA*	60.7 ± 4.9	39.3 ± 4.9	99.0 ± 0.8	1.0 ± 0.8
Δ*matAB*	81.1 ± 2.7	18.9 ± 2.7	84.0 ± 0.6	16.0 ± 0.6

Values represent the average participation fractions and standard deviations of two independent biological replicates.
