# Supplementary material for: Aggregation of germlings is a major contributing factor towards mycelial heterogeneity of Streptomyces
Source: Sci Rep. 2016 May 31;6:27045. doi: 10.1038/srep27045 (PMC4886682; doi:10.1038/srep27045)
Supplement: Supplementary Information [file srep27045-s1.pdf]

Aggregation of germlings is a major contributing factor towards mycelial heterogeneity of *Streptomyces*

Boris Zacchetti[1], Joost Willemse[1], Brand Recter[2], Dino van Dissel[1], Gilles P. van Wezel[1] H.A.B. Wösten[2, #] and Dennis Claessen[1,#]

1. Microbial Biotechnology, Institute of Biology, Leiden University, Sylviusweg 72, 2333 BE Leiden, The Netherlands

2. Microbiology, Department of Biology, Utrecht University, Padualaan 8, 3584 CH Utrecht, The Netherlands

# Correspondence should be addressed to H.A.B.W. (email: h.a.b.wosten@uu.nl) or D.C. (email: D.Claessen@biology.leidenuniv.nl)

Supplementary Information

Supplementary Table S1. Strains used in this study.

***Escherichia coli* strains**	**Description**	**Reference**
DH5α	F- Φ80*lac*ZDM15 D(*lacZYA-argF*)U169 *recA1 endA1 hsdR17*(rK-, mK-) *phoA supE44 thi-1 gyrA96 relA1* λ-	1
ET12567	F-*dam-13::*Tn9 *dcm-6 hsdM hsdR recF143 zjj-202::*Tn10 *galK2 galT22 ara14 lacY1 xyl-5 leuB6 thi-1 tonA31 rpsL136 hisG4 tsx78 mtl-1 glnV44*	2
***Streptomyces* strains**		
S. coelicolor A3(2) M145	Wild-type *SCP1*$^-$ *SCP2*$^-$	Laboratory stock
S. lividans 1326	Wild-type	Laboratory stock
S. scabies ISP5078	Wild-type	Gift from Prof. Loria
S. albus	Wild-type	Laboratory stock
S. lividans Δ*cslA*	*S. lividans* 1326 lacking *cslA* (marker-less)	3
S. lividans Δ*glxA*	*S. lividans* 1326 lacking *glxA* (marker-less)	3
S. lividans Δ*matAB*	*S. lividans* 1326 lacking *matA* and *matB* (marker-less)	4
S. coelicolor M512	*Streptomyces coelicolor* A3(2) M145 lacking *redD* and *actII-ORF4*	5
S. lividans pGreen	*S. lividans* 1326 containing pGreen	This work
S. lividans pRed	*S. lividans* 1326 containing pRed	This work
S. lividans Δ*cslA* pGreen	*S. lividans* Δ*cslA* containing pGreen	This work
S. lividans Δ*cslA* pRed	*S. lividans* Δ*cslA* containing pRed	This work
S. lividans Δ*glxA* pGreen	*S. lividans* Δ*glxA* containing pGreen	This work
S. lividans Δ*glxA* pRed	*S. lividans* Δ*glxA* containing pRed	This work
S. lividans Δ*matAB* pGreen	*S. lividans* Δ*matAB* containing pGreen	This work
S. lividans Δ*matAB* pRed	*S. lividans* Δ*matAB* containing pRed	This work
S. lividans pGreen pRed	*S. lividans* 1326 containing pGreen and pRed	This work
S. coelicolor M512 pGreen	*S. coelicolor* M512 containing pGreen	This work
S. scabies ISP5078 pRed	*S. scabies* ISP5078 containing pRed	This work

Supplementary Table S2. Vectors and constructs used in this study.

**Name**	**Description and relevant features**	**Reference**
pGreen	pIJ8630 containing *eGFP* under control of the constitutive *gap1* promoter of *S. coelicolor* A3(2) M145.	This work
pRed	pMS82 containing *mCherry* under control of the constitutive *gap1* promoter of *S. coelicolor* A3(2) M145.	This work
pIJ8630	*E. coli–Streptomyces* shuttle vector containing the φC31 *attP-int* region for genomic integration. Contains an apramycin resistance cassette.	6
pMS82	*E. coli–Streptomyces* shuttle vector containing the φBT1 *attP-int* region for genomic integration. Contains an hygromycin resistance cassette.	7
pIJ2925	Derivative of pUC18 that contains two BglII sites flanking a modified MCS derived from plasmid pIJ486	8
pRSET-B	Plasmid containing the *mCherry* gene	R. Tsien

Supplementary Table S3. Primers used in this study.

Primer name	Sequence 5'-3'	Restriction site
Gap1-FW	GATAGATCTCCGAGGGCTTCGAGACC	BglII
Gap1-RV	GCCCATATGCCGATCTCCTCGTTGGTACG	NdeI
Gap1-FW*	AAAGGTACCACGCAGACCGAGGGCTTCGAG	KpnI
mCherry-FW	TAACATATGGTGAGCAAGGGCGAGGAGGATAAC	NdeI
mCherry-RV	GGGAAGCTTTTACTTGTACAGCTCGTCCATGC	HindIII
RT-cslA-FW	AGTCGCAGCAGTTCCTCTTC	
RT-cslA-RV	TTCTTGTGGCGGTGCATCTC	
RT-glxA-FW	AGTTCGAGCAGCGGATCGAG	
RT-glxA-RV	TCAGCCGCACCTTCTTGACC	
RT-matA-FW	CTCGGAGGCTGGACGAGATG	
RT-matA-RV	GGCCGCCTATTCGGGAAC	
RT-matB-FW	AGTCCGAGAAGCGCATCGACTG	
RT-matB-RV	GTCCTCGCTGTCGGTGTTGTTG	
RRNA-1	AGAGTTTGATCCTGGCTCAG	
RRNA-2	CGAACCTCGCAGATGCCTG	

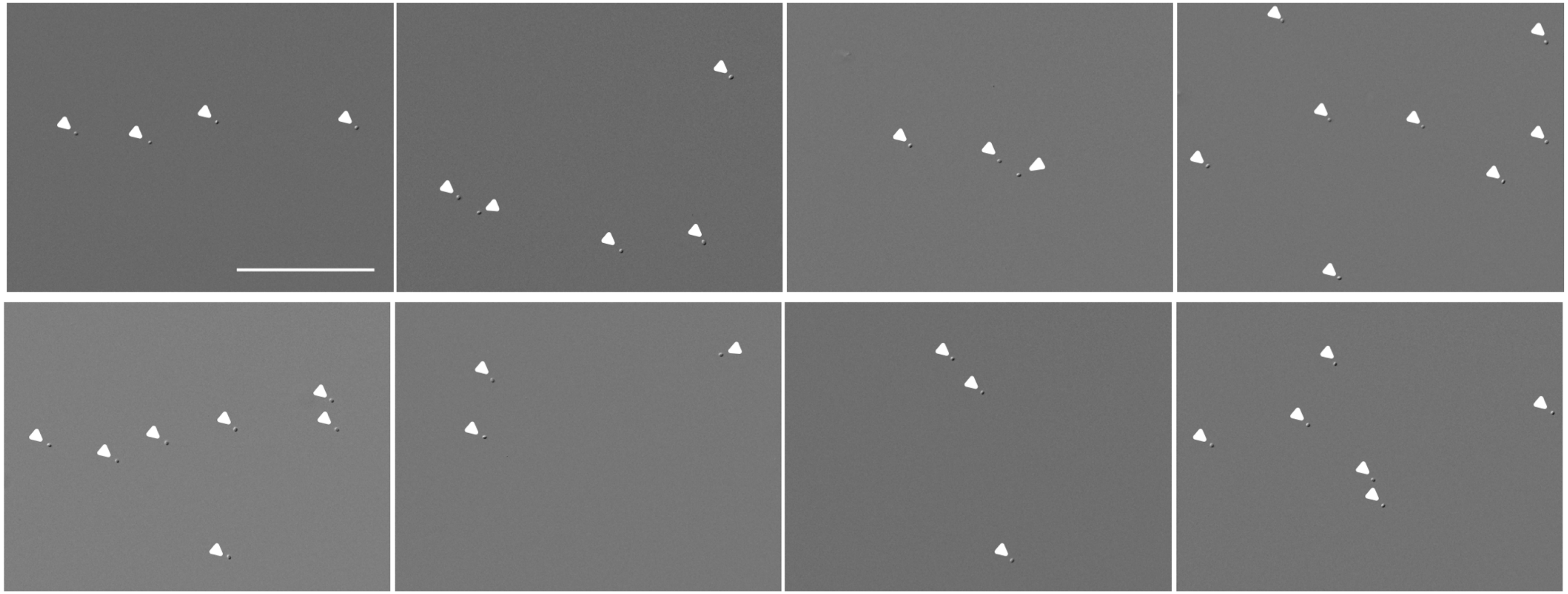

Figure S1. Collage of representative images of particles in TSBS cultures after 2 h of growth. The arrowheads indicate individual spores in the culture medium. Neither spore germination nor aggregation were detected at this time point. Scale bar represents 50 µm.

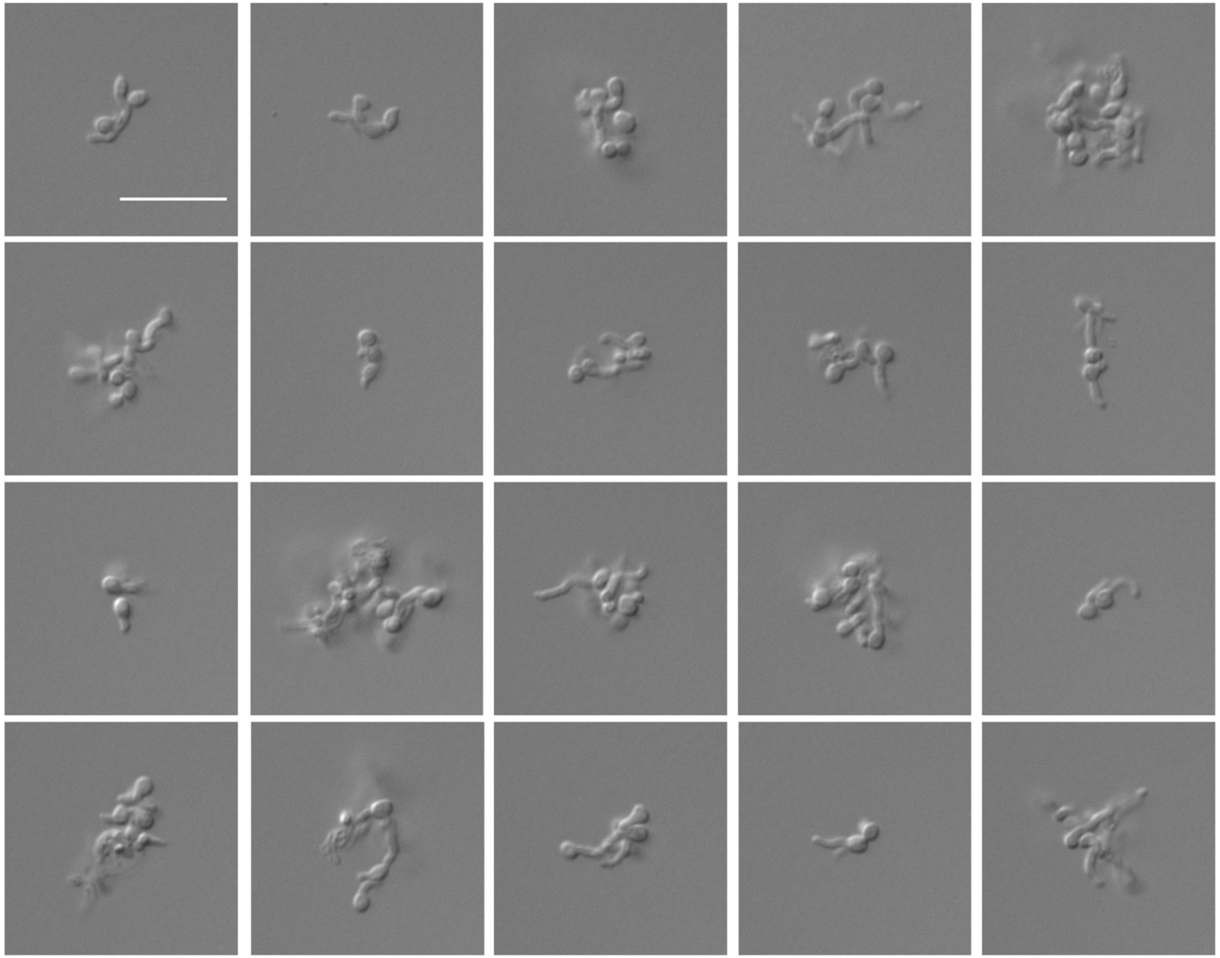

Figure S2. Collage of representative images of spore aggregates in TSBS cultures. Small aggregates invariably contain germlings. Pictures were taken after 5 h of growth. Scale bar represents 50 µm.

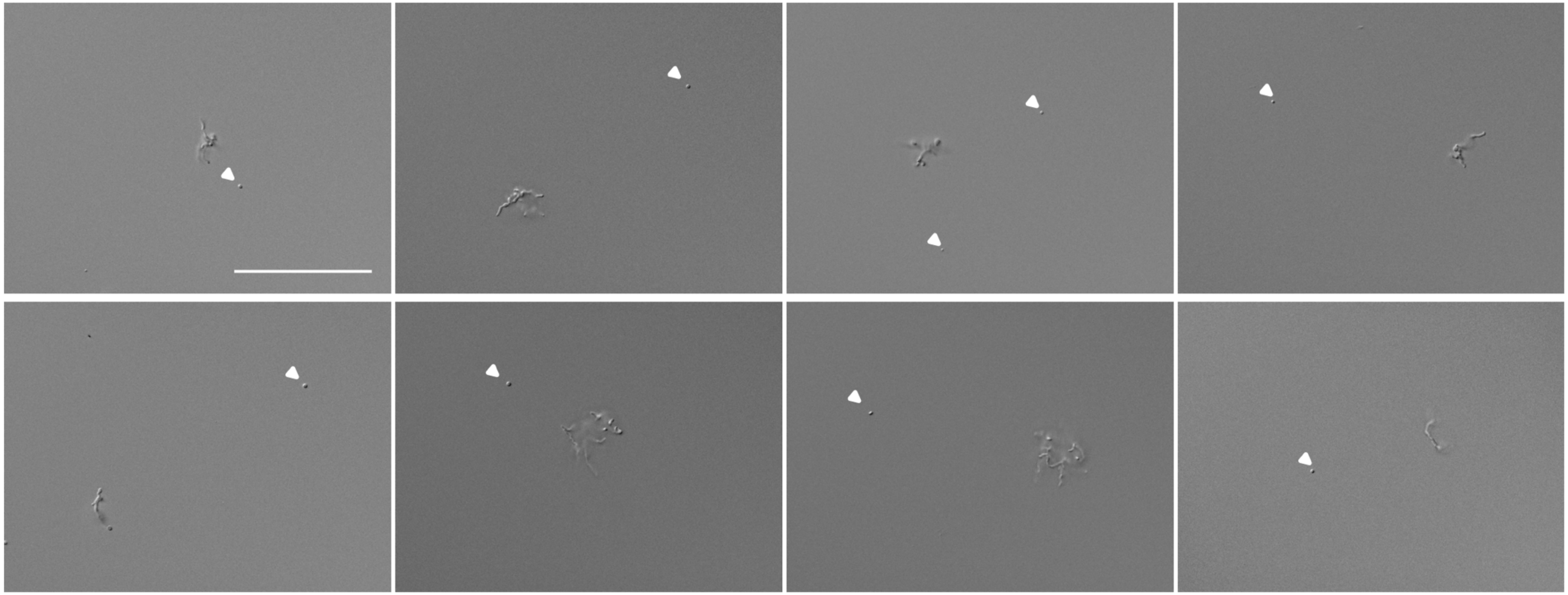

Figure S3. Collage of representative images of particles in TSBS cultures after 5 h of growth. In addition to small aggregates, individual spores (arrowheads) are visible. Please note that these spores show no visible germ tubes. Scale bar represents 50 µm.

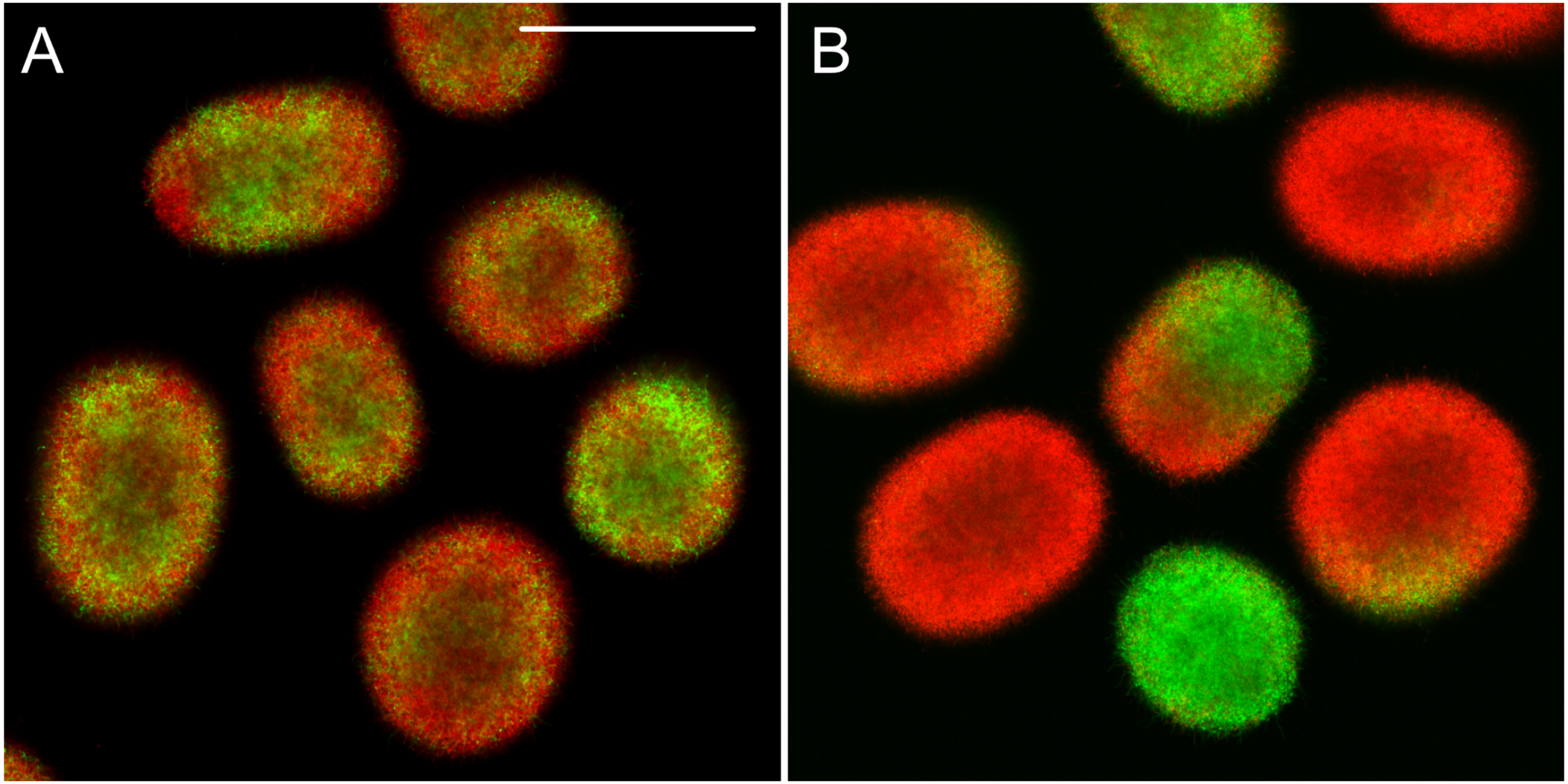

Figure S4. Fluorescent pellets of aggregated *Streptomyces lividans* strains. The strains expressing either the *eGFP* or the *mCherry* gene were grown separately for 2 (A) or 8 (B) hours prior to mixing them. Pictures of the aggregated strains were taken after 24 h of growth in NMMP medium. Scale bar represents 200 µm.

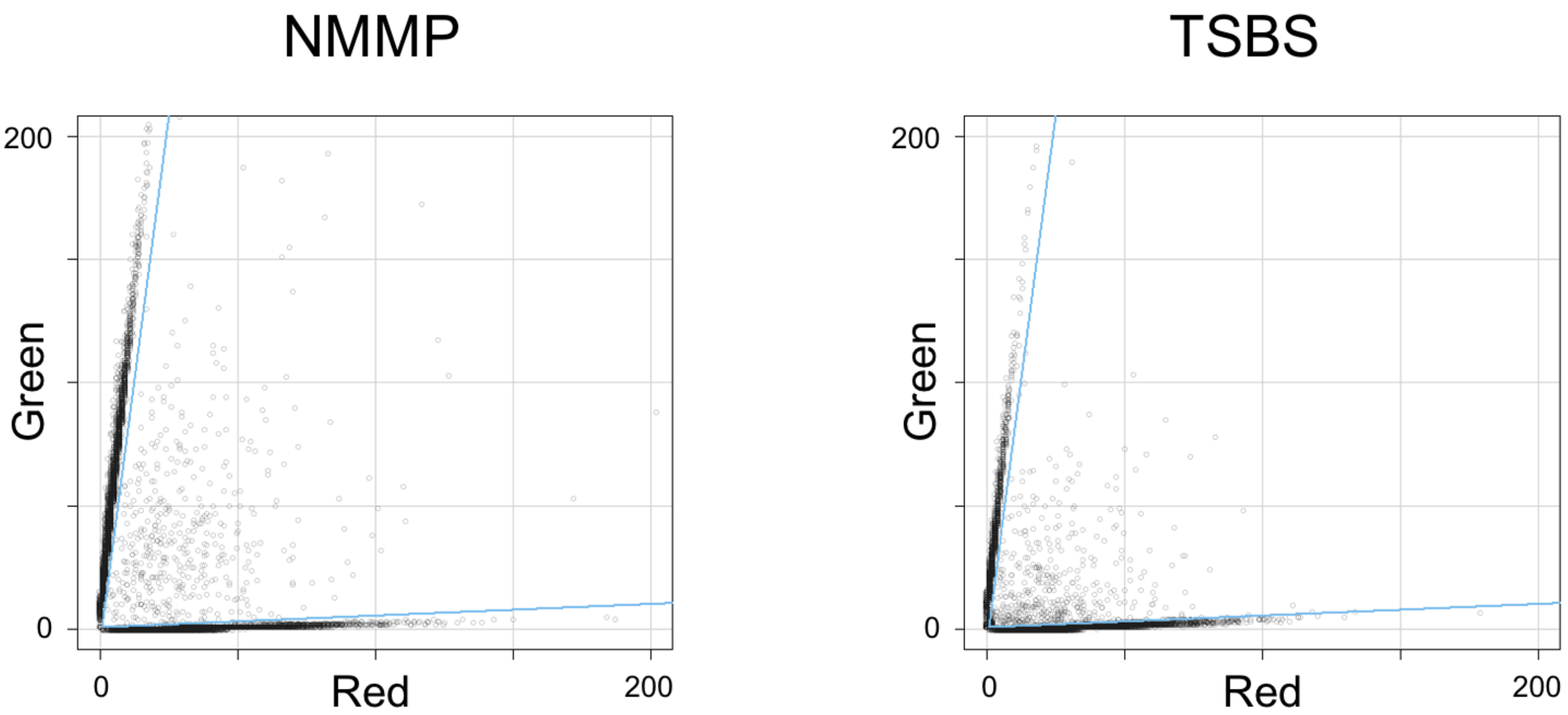

Figure S5. Time-dependent aggregation is not caused by secreted molecules accumulating in the medium. The strains expressing either the *eGFP* or the *mCherry* gene were grown separately for 12 hours, after which they were mixed in fresh NMMP (left) or TSBS (right) medium. Each plot represents the fluorescence intensities of pellets in the red (X-axis) and green (Y-axis) channel determined by particle analysis. Quantification indicates that 3.5% and 16.1% of the particles contain both fluorescences in NMMP and TSBS medium, respectively.

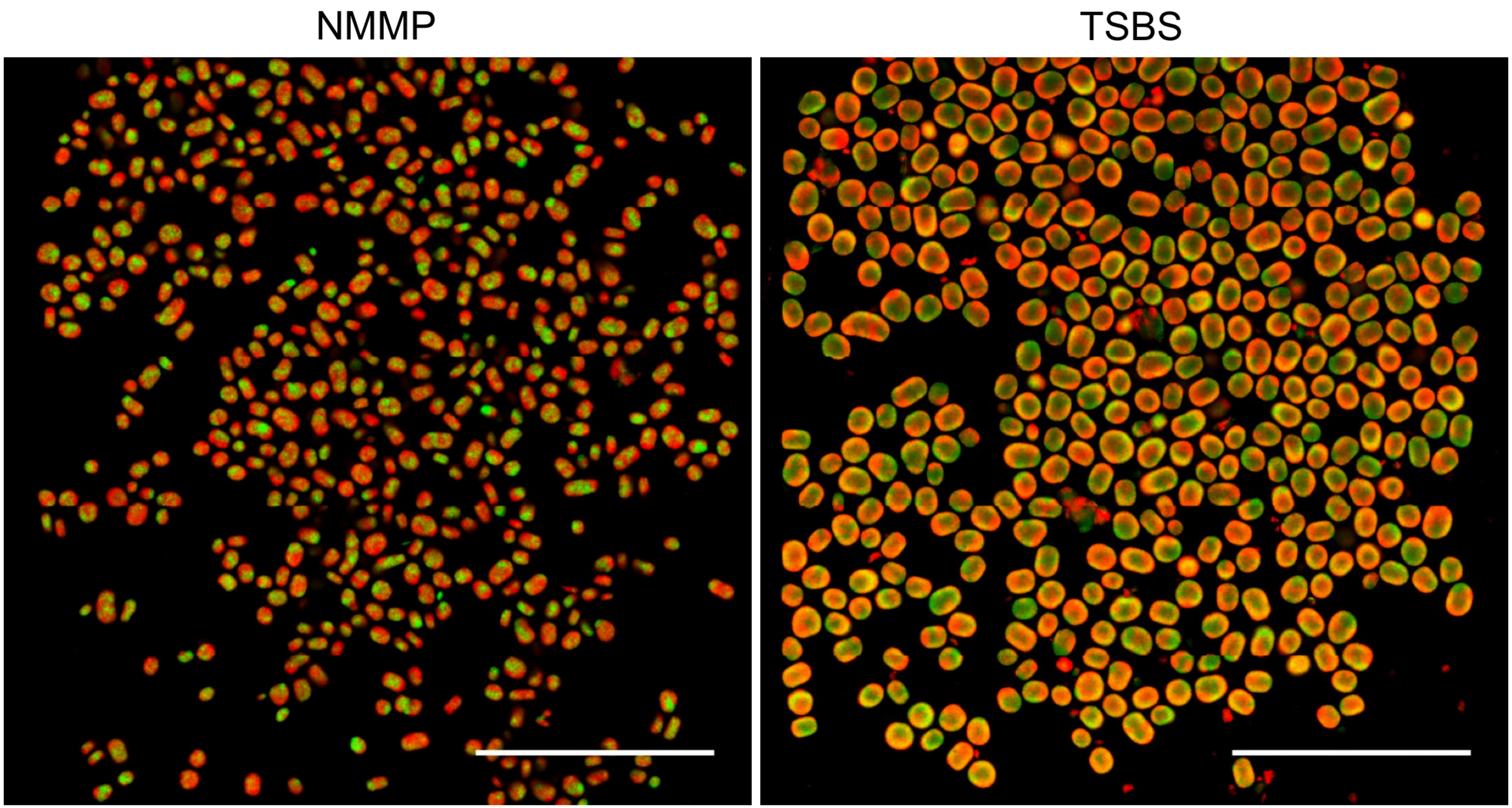

Figure S6. Analysis of aggregation of fluorescent wild-type strains. Spores of the wild-type strains expressing either the *eGFP* or the *mCherry* gene were mixed at the onset of growth in NMMP (left) or TSBS (medium). After 24 h of growth, most pellets are composed of both types of fluorescent hyphae. Scale bar represents 2 mm.

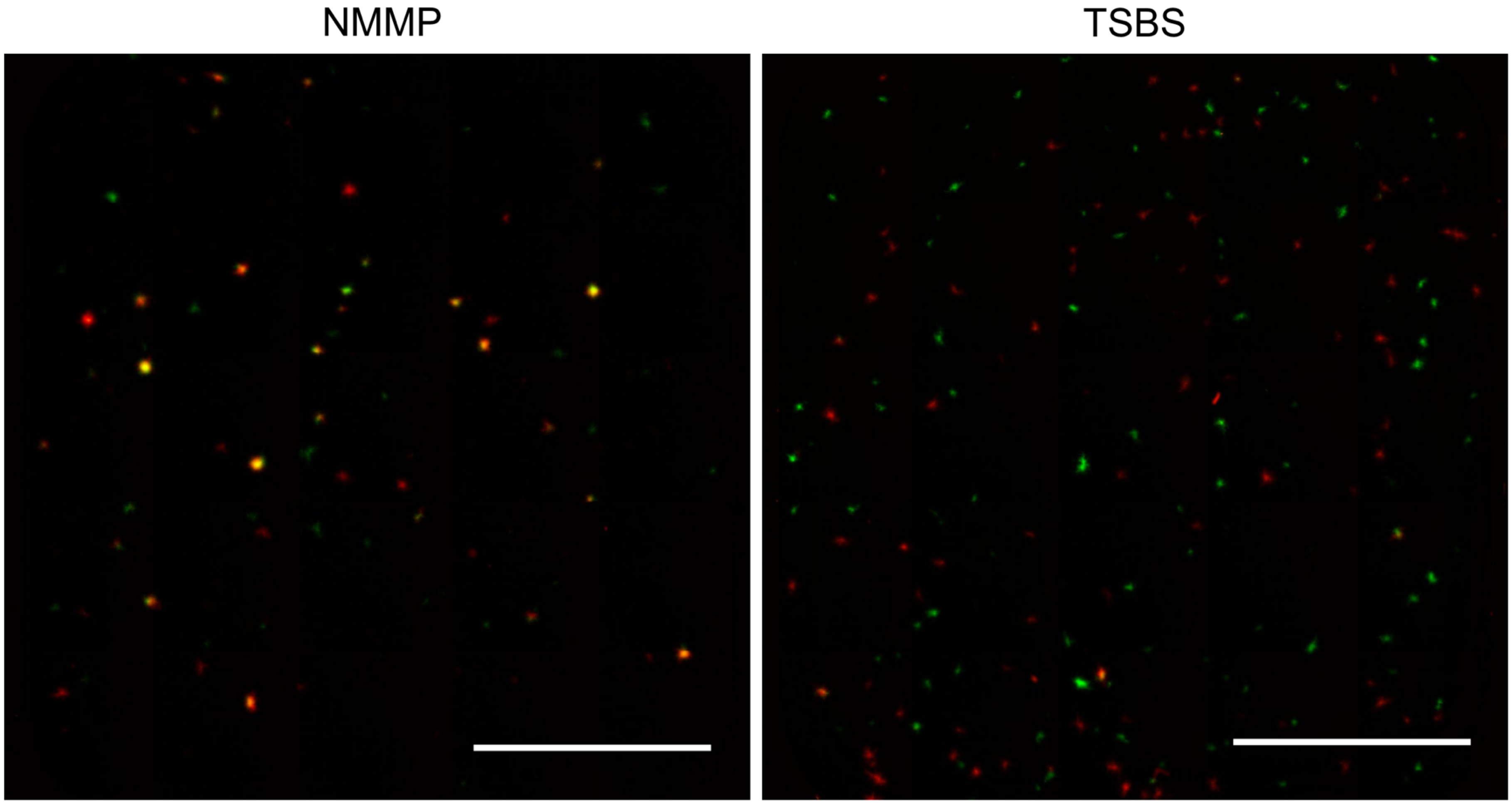

Figure S7. Aggregation depends on CslA. Spores of the Δ*cslA* derivative strains expressing either the *eGFP* or the *mCherry* gene were mixed at the onset of growth in NMMP (left) or TSBS (medium). After 24 h of growth, the majority of particles is either green or red fluorescent in the absence of *cslA*. Scale bar represents 2 mm.

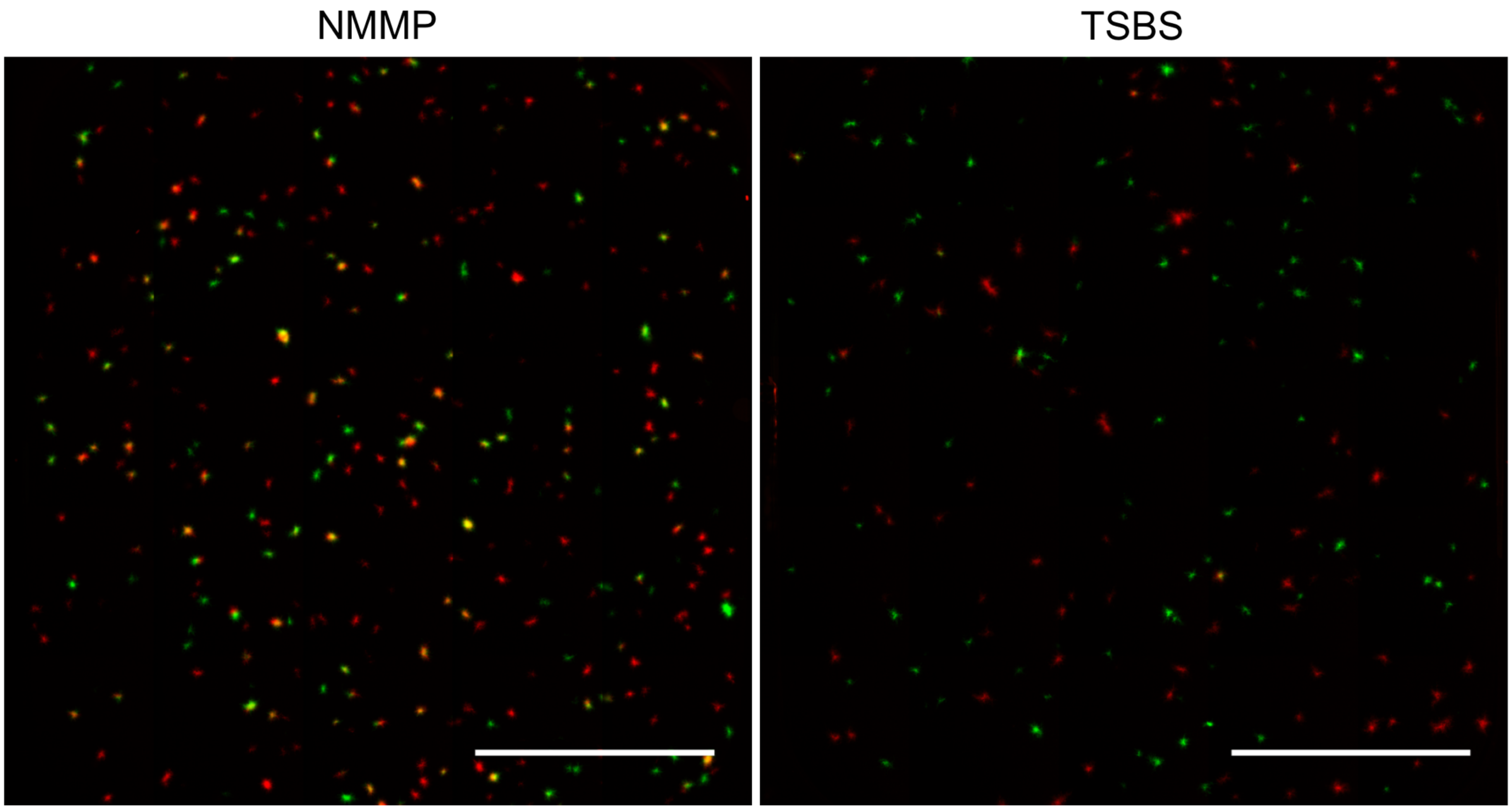

Figure S8. Aggregation depends on GlxA. Spores of the Δ*glxA* derivative strains expressing either the *eGFP* or the *mCherry* gene were mixed at the onset of growth in NMMP (left) or TSBS (medium). After 24 h of growth, most particles are either green or red fluorescent in the absence of *glxA*. Scale bar represents 2 mm.

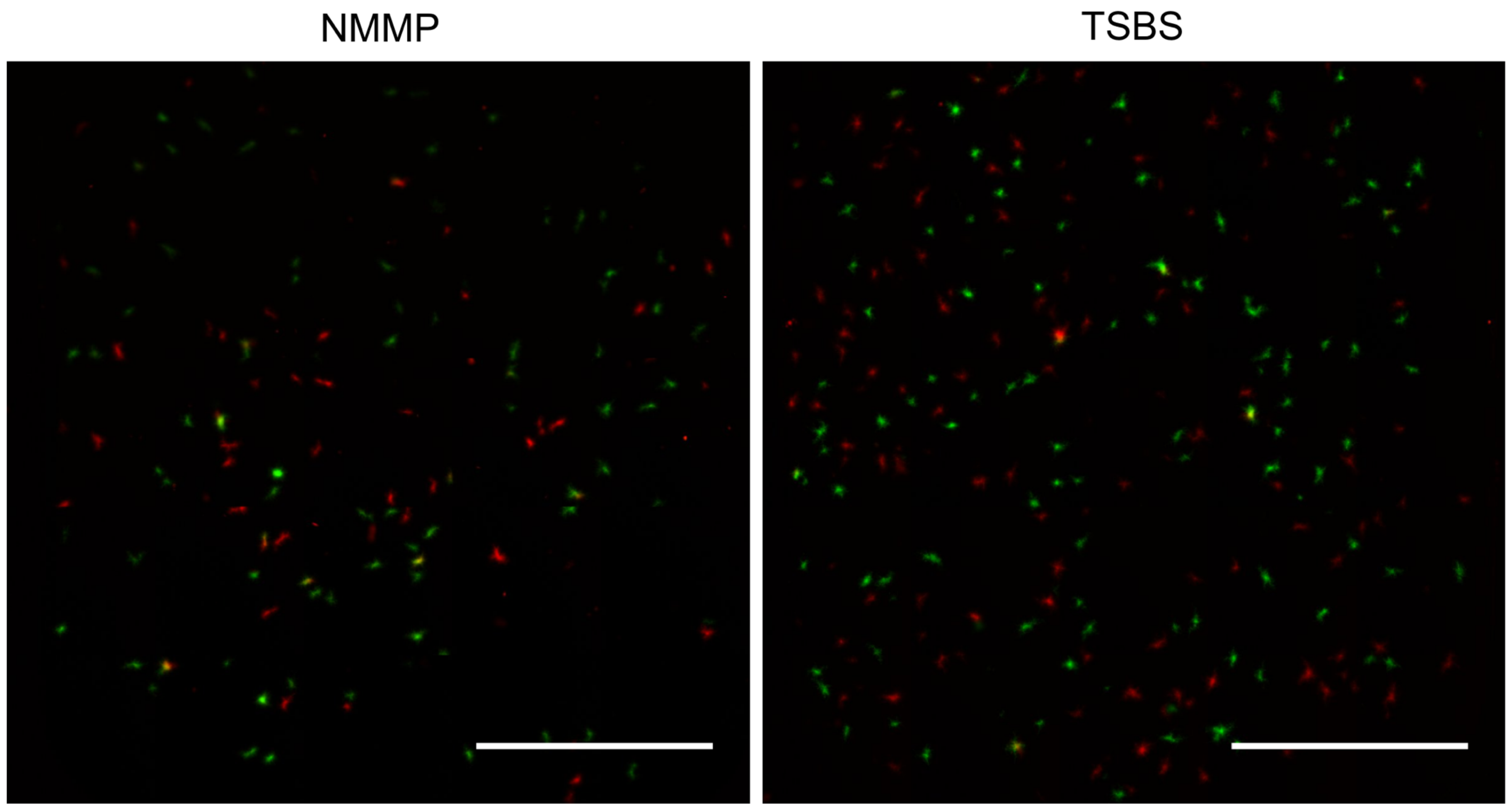

Figure S9. Aggregation depends on MatAB. Spores of the Δ*matAB* derivative strains expressing either the *eGFP* or the *mCherry* gene were mixed at the onset of growth in NMMP (left) or TSBS (medium). After 24 h of growth, the majority of particles is either green or red fluorescent in the absence of the *matAB* genes. Scale bar represents 2 mm.

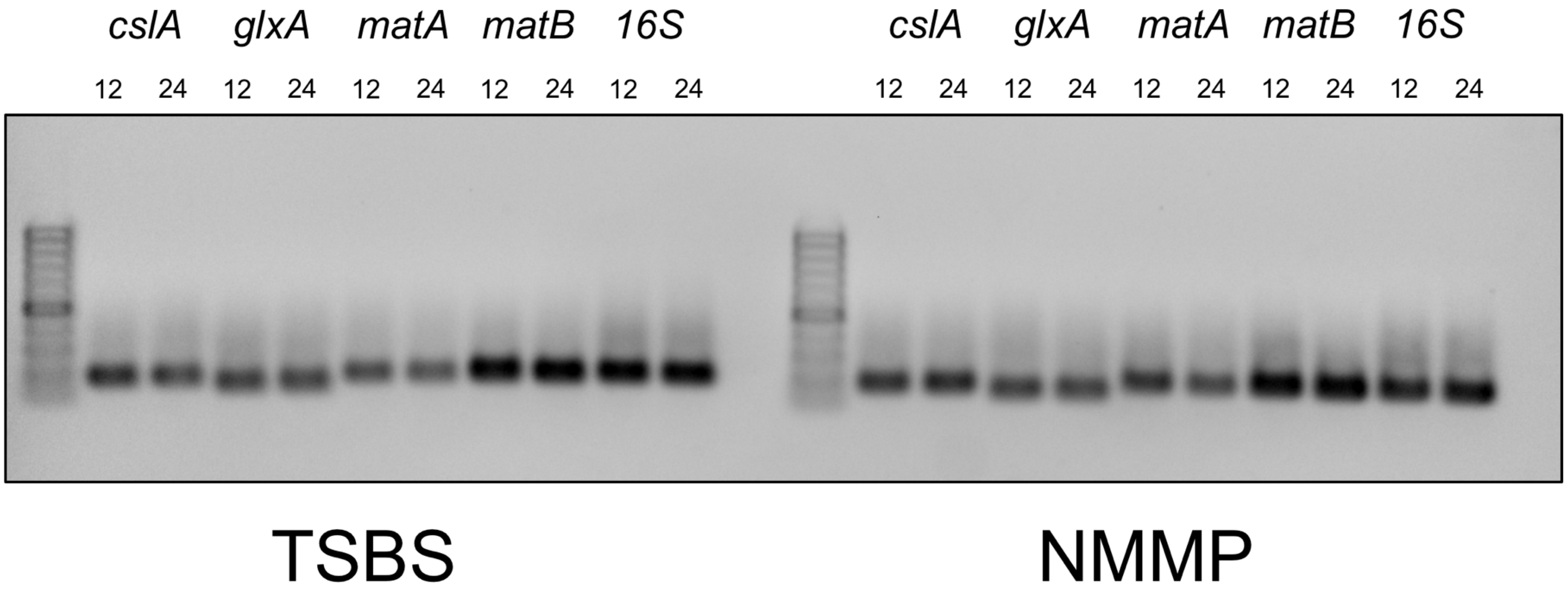

Figure S10. Genes required for glycan biosynthesis are expressed in NMMP and TSBS medium. Total RNA was isolated from NMMP and TSBS cultures after 12 and 24 hours of growth and analysed for the presence of transcripts of the *cslA, glxA, matA* and *matB* genes using RT-PCR. *16S rRNA* was used as a control. Note that all genes are expressed at both time points in both media.

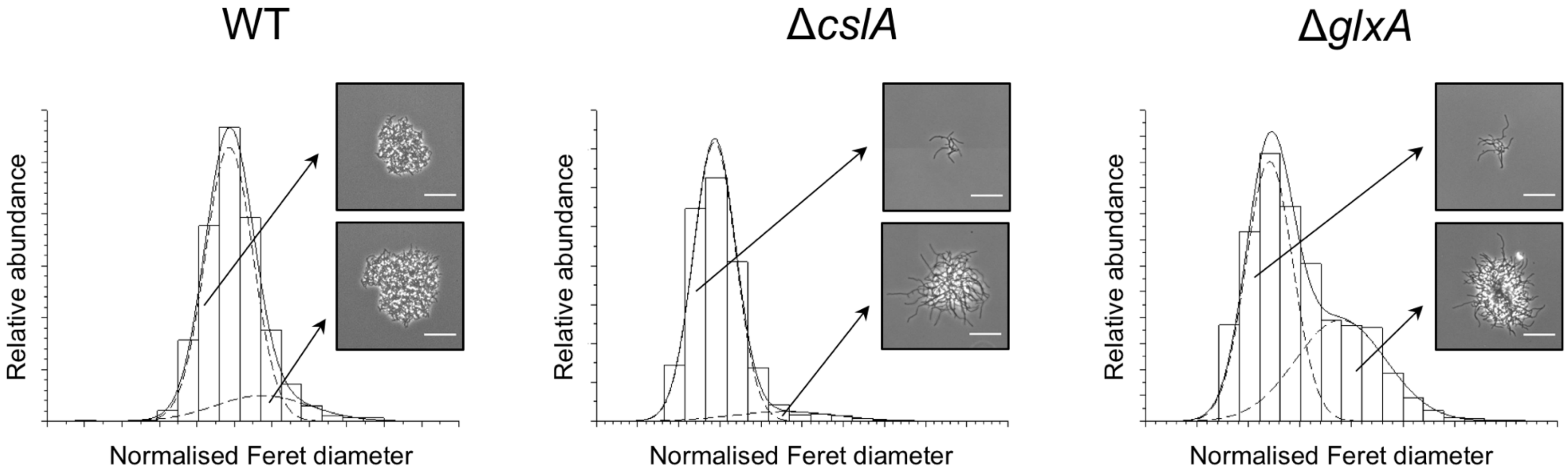

Figure S11. Particle size distributions of the wild-type, Δ*cslA* and Δ*glxA* strains related to mycelial morphology. After 12 h of growth in NMMP medium, particles of the wild-type (left) and Δ*cslA* strains (middle) are more homogenous in size than those of the Δ*glxA* strain (right). Note that most particles of the Δ*cslA* strain are small and characterized by an open morphology, while the wild-type strain almost exclusively forms pellets that are larger and denser. In the Δ*glxA* strain both types of mycelia are present and which are more equally distributed. Scale bars represent 25 µm.

References

1 Hanahan, D. Studies on transformation of *Escherichia coli* with plasmids. *J Mol Biol* **166**, 557-580 (1983).

2 MacNeil, D. J. *et al.* Analysis of *Streptomyces avermitilis* genes required for avermectin biosynthesis utilizing a novel integration vector. *Gene* **111**, 61-68 (1992).

3 Chaplin, A. K. *et al.* GlxA is a new structural member of the radical copper oxidase family and is required for glycan deposition at hyphal tips and morphogenesis of *Streptomyces lividans*. *Biochem J* **469**, 433-444, doi:10.1042/BJ20150190 (2015).

4 van Dissel, D., Claessen, D., Roth, M. & van Wezel, G. P. A novel locus for mycelial aggregation forms a gateway to improved *Streptomyces* cell factories. *Microb Cell Fact* **14**, 44, doi:10.1186/s12934-015-0224-6 (2015).

5 Floriano, B. & Bibb, M. J. *afsR* is a pleiotropic but conditionally required regulatory gene for antibiotic production in *Streptomyces coelicolor* A3(2). *Mol Microbiol* **21**, 385-396 (1996).

6 Sun, J., Kelemen, G. H., Fernández-Abalos, J. M. & Bibb, M. J. Green fluorescent protein as a reporter for spatial and temporal gene expression in *Streptomyces coelicolor* A3(2). *Microbiology* **145**, 2221-2227 (1999).

7 Gregory, M. A., Till, R. & Smith, M. C. M. Integration site for *Streptomyces* phage phiBT1 and development of site-specific integrating vectors. *J Bacteriol* **185**, 5320-5323 (2003).

8 Janssen, G. R. & Bibb, M. J. Derivatives of pUC18 that have *Bgl*II sites flanking a modified multiple cloning site and that retain the ability to identify recombinant clones by visual screening of *Escherichia coli* colonies. *Gene* **124**, 133-134 (1993).
